# Multi-omics and spatial transcriptomics identify KCMF1 as an immune-metabolic driver of hepatocellular carcinoma progression

**DOI:** 10.1007/s12672-026-05188-6

**Published:** 2026-05-12

**Authors:** Zhan Liu, Saobo Wu, Yinshi Li, Chenhong Zhou, Yifei Liu, Yanqun Guan

**Affiliations:** 1 Xinjiang Second Medical College, Karamay, China; 2 Xi’an Jiaotong University, Xi’an, China; 3 Xinjiang Medical University, Urumqi, China

**Keywords:** Hepatocellular carcinoma, Ubiquitination, KCMF1, Tumor microenvironment, Prognostic biomarker

## Abstract

Hepatocellular carcinoma (HCC) is a leading cause of cancer mortality, characterized by poor prognosis and limited treatment options. Ubiquitination, a key post-translational modification, plays crucial roles in cancer progression. However, the specific contribution of ubiquitination-associated genes to HCC remains largely unexplored. Public datasets from TCGA and GEO were analyzed to identify ubiquitination-associated genes (K48 and K63 ubiquitin chains). Prognostic models were constructed using multiple regression algorithms, consistently identifying KCMF1 as the top contributor. Bulk transcriptomic analysis was performed to assess differential expression, immune infiltration, and pathway enrichment. Single-cell and spatial transcriptomic analyses further investigated KCMF1’s expression and its functional role in the tumor microenvironment. KCMF1 was significantly overexpressed in HCC tissues and strongly associated with poor overall and disease-specific survival. Transcriptomic analysis revealed its positive correlation with immune cell populations enriched in the tumor microenvironment, such as M0 macrophages (naive/precursor macrophages that serve as the cellular source for TAM polarization). Functional enrichment highlighted KCMF1’s involvement in VEGF and TGFβ signaling pathways, which promote angiogenesis and immune suppression. Single-cell analysis identified KCMF1-positive malignant cells as hubs of intercellular communication, while spatial transcriptomics confirmed its enrichment in malignant regions, underscoring its role in tumor-stroma interactions. KCMF1 is a pivotal ubiquitination-associated gene that drives HCC progression through immune modulation. Integrative single-cell and spatial transcriptomics further locate KCMF1-enriched malignant niches, providing spatially resolved biomarker evidence. Its consistent prognostic relevance across analyses highlights its potential as a biomarker and therapeutic target. Therefore, KCMF1-directed therapy and combined immune-metabolic interventions warrant further investigation.

**Supplementary Information:**

The online version contains supplementary material available at 10.1007/s12672-026-05188-6.

## Introduction

Hepatocellular carcinoma (HCC) is one of the leading causes of cancer-related mortality worldwide, characterized by a high incidence rate and poor prognosis [1]. Despite advancements in immunotherapy and targeted therapies, such as those targeting the programmed cell death protein 1 (PD-1) pathway and its ligand PD-L1, the overall prognosis for HCC remains unfavorable [2, 3]. More than 70% of patients who undergo surgical resection or ablation experience tumor recurrence within five years, reflecting the aggressive nature of this disease [4]. These challenges, compounded by late-stage diagnosis and the limited efficacy of existing treatment strategies, highlight the urgent need for novel diagnostic and therapeutic approaches [5, 6].

Ubiquitination, a critical post-translational modification, plays a vital role in regulating protein stability, activity, and localization, thereby maintaining cellular homeostasis [7–9]. In our previous study, we found that LIMA1 plays an important role in liver cancer through ubiquitination modification. As an E3 ligase, RNF40 can ubiquitinate LIMA1 and promote its degradation, thereby regulating liver cancer cell proliferation and lipid synthesis metabolism. Therefore, we hypothesize that ubiquitination regulation may occupy a unique position in the development and progression of liver cancer. Among the various types of polyubiquitin chains, those linked through lysine 48 (K48) and lysine 63 (K63) are the most extensively studied. K48-linked ubiquitination primarily targets proteins for proteasomal degradation, ensuring proper protein turnover. In contrast, K63-linked ubiquitination does not direct proteins for degradation but instead regulates non-proteolytic functions, such as intracellular signaling, immune responses, and error-free DNA postreplication repair [10, 11]. Dysregulation of these pathways is closely associated with cancer progression, including disruptions in the cell cycle, DNA repair mechanisms, and immune regulation [12–14].

Although the functions of K48- and K63-linked ubiquitin chains and their roles in tumor biology are well-documented, systematic studies on the regulatory genes governing these processes in HCC remain limited [15–17]. Particularly, their relevance to HCC prognosis and their potential as therapeutic targets have not been systematically explored. Further investigation into these genes may provide novel insights into the molecular mechanisms underlying HCC progression and contribute to the development of new diagnostic and therapeutic targets.

This study utilizes K48- and K63-linked ubiquitin chain-associated genes to develop a prognostic model for HCC. The top prognostic gene was identified and further analyzed to elucidate its role in tumor progression and its potential as a biomarker and therapeutic target. Using genes associated with ubiquitination processes as a starting point, this study provides insights into HCC progression and lays the groundwork for innovative diagnostic and therapeutic strategies.

## Methods

### Data acquisition and preparation

A comprehensive collection of publicly available datasets was utilized in this study to perform pan-cancer and HCC-specific analyses. For the pan-cancer analysis, RNA sequencing and clinical data were sourced from the TCGA database (https://portal.gdc.cancer.gov/) and multiple GEO cohorts. Specifically, GEO datasets such as GSE9893, GSE87211, GSE181063, GSE21846, GSE69053, E-TABM-898, GSE33331, CGGA-693, GSE76427, GSE72094, GSE190266, GSE42127, GSE102073, GSE17260, GSE32062, E-MTAB-6134, GSE116918, GSE54460, and GSE70769 were retrieved from the GEO database (https://www.ncbi.nlm.nih.gov/geo/). These datasets, comprising RNA sequencing data and associated clinical information, were filtered to exclude non-tumor samples and those lacking survival data. To ensure consistency in downstream analyses, Ensembl IDs were converted to gene symbols.

To construct the HCC prognostic model and investigate the association between specific genes and HCC progression, additional datasets were incorporated. RNA sequencing and clinical data for HCC patients were retrieved from the TCGA-LIHC cohort, as well as GEO datasets, including GSE116174, GSE144269, GSE14520, GSE54236, and GSE76427. Data from the ICGC-LIRI cohort (https://dcc.icgc.org/) were also included. Samples lacking survival data or classified as non-tumor were excluded, and all gene annotations were standardized to gene symbols.

Single-cell transcriptomic data were incorporated to explore cellular heterogeneity and microenvironmental dynamics. Datasets GSE166635, GSE146115, and GSE146409 were downloaded from the GEO database. Preprocessing included quality control, cell clustering, differential expression analysis, cell type annotation, and malignant cell classification, following the TISCH workflow [18].

Spatial transcriptome data were used to integrate spatial information with transcriptomic profiles. These datasets were obtained from publicly available resources linked to PMID: 36,708,811 and accessed through Mendeley Data (identifier: skrx2fz79n). The dataset includes two HCC samples, designated as HCC1 and HCC2, corresponding to P15T and P3T in the Mendeley database. Spatial transcriptome data underwent quality control and spatial mapping for downstream analyses [19].

All data analyzed in this study were derived from publicly accessible resources, including previous publications and established databases.

### Machine learning model construction and gene selection

The K48 and K63 ubiquitin chain-associated gene sets were obtained from the MSigDB database (https://www.gsea-msigdb.org/gsea/msigdb). An intersection analysis was performed to identify a candidate gene set, referred to as the Intersection Gene Set (IGS), which was used as the foundation for constructing a cancer gene expression prognostic model. Non-tumor samples and those with missing values were excluded to ensure data quality. Survival time was converted from days to years, and z-score normalization was applied to standardize gene expression data in validation datasets, ensuring a mean of 0 and variance of 1.

To balance model accuracy and interpretability, the genes from the IGS were utilized as input features for linear model construction without additional selection or dimensionality reduction, following established practices. A linear model was chosen for its simplicity and the ability to clearly attribute the contribution of each gene to prognosis. We employed an integrative machine learning pipeline to identify the most robust prognostic signature. Specifically, we constructed and evaluated models using distinct algorithms: Lasso, Ridge, Elastic Net (with α parameters ranging from 0.1 to 0.9), Stepwise Cox regression (both forward and backward selection), and CoxBoost. Specifically, for LASSO/Elastic Net, we specified the cross-validation folds (nfolds = 10), the loss metric (type.measure ="deviance”), and the criteria for lambda selection (λ.min).

Lasso regression was implemented using the ‘glmnet’ package, with the family parameter set to ‘cox’ and the alpha parameter fixed at 1. Ten-fold cross-validation was conducted using the cv. glmnet function to determine the optimal λ value, and non-zero coefficients corresponding to the optimal λ were extracted to identify significant genes. Elastic Net and Ridge regression were similarly implemented using the ‘glmnet’ package, with the alpha parameter for Elastic Net set between 0 and 1 (e.g., 0.1 to 0.9), and fixed at 0 for Ridge regression. Stepwise Cox regression was performed by first constructing multivariate Cox models using the coxph function, followed by stepwise selection with the stepAIC function, using both forward and backward selection. For CoxBoost, the penalty parameter was optimized with the optimCoxBoostPenalty function, and cross-validation using the cv.CoxBoost function was employed to determine the optimal number of steps. The final CoxBoost model was then constructed based on these parameters, and coefficients were extracted using the coef function.

Risk scores were calculated by combining model coefficients with gene expression data. Model performance was assessed through Receiver operating characteristic (ROC) curves and the area under the curve (AUC) at 1-, 3-, and 5-year time points using the ‘timeROC’ package. Kaplan-Meier survival analysis was performed with the ‘survminer’ package to stratify patients into high- and low-risk groups, ensuring group proportions were ≥ 0.3. Statistical significance between survival curves was determined using log-rank tests. Gene expression patterns and risk group distributions were visualized with the ‘ComplexHeatmap’ package. Univariate Cox analysis was conducted to compute hazard ratios (HR) for risk scores, and meta-analysis using the inverse variance method was applied to evaluate the prognostic value across datasets.

### Gene expression analysis

Gene expression data were standardized to facilitate both pan-cancer and tumor-specific analyses. For pan-cancer analyses, Z-scores (x − µ)/σ were calculated for each tumor type, and outliers (Z > 3 or Z < − 3) were excluded. Only tumor types with at least three normal samples after outlier removal were included. Differential expression between tumor and normal tissues was assessed using Wilcoxon Rank Sum Tests. For the LIHC cohort, single-tumor analyses were conducted after normalizing expression values using an upper quartile adjustment (set to 1000), followed by Z-score standardization. Both paired and unpaired differential analyses were performed using Wilcoxon Signed Rank Tests for paired tumor and adjacent normal tissues, and Wilcoxon Rank Sum Tests for unpaired comparisons. Given the established association of LIMA1 with HCC, correlation analysis between LIMA1 and the target gene was performed using TCGA data, providing further insight into their potential interplay in HCC [20].

To evaluate the diagnostic performance of gene expression profiles, ROC analysis was performed using the ‘pROC’ package. Metrics such as the AUC, 95% confidence intervals (CIs), and smoothed ROC curves were calculated to comprehensively assess discriminatory power.

### Survival prognosis analysis

Survival analysis was conducted to assess the prognostic significance of gene expression. Univariate Cox proportional hazards regression models were applied using the ‘survival’ package in R, with HRs and 95% CIs computed. The same methodology was used for external validation with GEO datasets. Kaplan-Meier survival analysis was performed to compare survival between high- and low-expression groups, with optimal expression cutoffs determined using the ‘survminer’ package, ensuring a minimum group size ratio of 0.3. Statistical significance was evaluated using log-rank tests. For HCC, univariate Cox analysis results were further integrated through meta-analysis using the inverse variance method, focusing on log-transformed HR values. Genes with HR < 1 were classified as tumor suppressors, while those with HR > 1 were classified as oncogenes. Multivariate Cox regression was conducted to evaluate the combined effects of gene expression and clinical variables, with results visualized using the ‘forestploter’ package.

To further investigate the relationship between LIMA1 and the target gene with HCC prognosis, the expression levels of the two genes were z-score normalized. Groups were defined as follows: z-score ≤ 0 indicated low expression, and z-score > 0 indicated high expression. Based on this, four subgroups were created: (1) High expression of both genes: LIMA1+ & target gene+; (2) Low expression of LIMA1 and high expression of the target gene: LIMA1- & target gene+; (3) Low expression of both genes: LIMA1- & target gene-; (4) High expression of LIMA1 and low expression of the target gene: LIMA1+ & target gene-. Kaplan-Meier survival analysis was performed using the ‘survival’ package in R, with the survfit function used for the log-rank test to evaluate the significance of overall and pairwise comparisons among the four groups.

### Genetic alteration analysis

Somatic single-nucleotide variant (SNV) data from 8,663 samples across 33 cancer types were obtained from the TCGA database. Mutation types included missense mutations, nonsense mutations, frameshift insertions and deletions, splice site mutations, and others. Non-coding mutations (e.g., Silent, Intron, 3’ UTR, 5’ UTR, 3’ Flank, and 5’ Flank) were excluded for mutation frequency calculations. The mutation frequency of each gene’s coding region was computed as: Mutation Frequency = Number of Mutated Samples / Total Number of Samples SNV oncoplots were generated using the ‘maftools’ package to visualize mutation distributions.

### Tumor microenvironment (TME) analysis

Immune infiltration analysis was conducted on bulk transcriptome data using algorithms such as ssGSEA, xCell, and CIBERSORT [21, 22]. For CIBERSORT, we used the reference signature (LM22), the number of permutations (e.g., 1000), and the quantile normalization settings. Correlations between gene expression and aspects of the immune microenvironment, including immune cell composition, immune molecules, immune response states, and immune scores, were quantified in the TCGA cohort [23–25]. Patients were stratified into four quartile groups (Q1–Q4) based on gene expression levels, with Q1 representing the highest 25% and Q4 the lowest 25%. Average scores for each group were calculated (excluding missing values) and visualized using the ‘pheatmap’ package.

### Enrichment analysis of the key gene

Enrichment analysis included Gene Ontology (GO) and Kyoto Encyclopedia of Genes and Genomes (KEGG) pathway analyses, as well as Gene Set Enrichment Analysis (GSEA) [26–28]. For GO and KEGG analyses, the TCGA-LIHC cohort was divided into high- and low-expression groups based on the median expression level of the key gene. Differentially expressed genes (DEGs) were identified using the ‘limma’ package with thresholds of logFC > 1 and *P* < 0.05. These DEGs were then used to identify enriched biological processes and pathways. For GSEA, the entire gene expression dataset was analyzed without additional filtering, allowing a broader exploration of enriched pathways.

Building on the enrichment analysis, the CancerSEA database was utilized to investigate 14 functional states of tumor cells. Pathway activity was quantified using the z-score algorithm proposed by Lee et al., with scores for the 14 functional state gene sets calculated using the ‘GSVA’ package [29]. These scores were normalized using the scale function, and Pearson correlation coefficients were computed to evaluate associations between gene expression and functional state scores.

### Single-cell RNA-seq analysis

Single-cell RNA-seq analysis was conducted using the ‘Seurat’ R package. Cells with gene expression levels outside 500–8000 or mitochondrial expression exceeding 15% were excluded. Data normalization was performed using SCTransform, followed by dimensionality reduction with PCA and batch effect correction with Harmony [30, 31]. Clustering was conducted using FindNeighbors and FindClusters, and results were visualized with Uniform Manifold Approximation and Projection (UMAP). Cell types were annotated using marker genes from the CellMarker database. UMAP visualized the expression of key gene, and the Kruskal-Wallis test assessed its variation across cell types. Cells were grouped into gene-positive and gene-negative, with proportions calculated for each cell type [18, 32]. Here, KCMF1-positive was defined as normalized expression above the median value of the given cell type.

Intercellular communication analysis was conducted using the ‘CellChat’ package (version 1.6.1). Communication networks were constructed to evaluate interaction frequency and strength across different cell types, including interactions between single cells and other cell populations. For each cell type, we calculated outgoing and incoming strength and identified key signaling pathways to understand the flow of communication. Additionally, we analyzed sources and targets within the networks to highlight important signaling hubs. Key results were visualized to compare communication patterns and pathway activity across cell types [33].

For pan-cancer analysis, Gene expression data at single-cell resolution across multiple cancer types were retrieved from the TISCH database. The ‘pheatmap’ package was used to construct heatmaps visualizing the pan-cancer single-cell expression landscape of the key gene. Hierarchical clustering was performed using Ward’s minimum variance method and Euclidean distance as the metric, facilitating the identification of patterns and trends in the data. This analysis helped assess the conservation of gene expression across different cell types and cancer contexts.

### Spatial transcriptomic analysis

Spatial transcriptomic analysis was conducted to investigate the spatial distribution and role of the key gene within the TME. To assess cellular composition at each spot on the 10x Visium slides, we used the ‘SPOTlight**’** package for deconvolution analysis. Rigorous quality control measures were applied to ensure data reliability, including checks on the number of expressed genes, unique molecular identifiers, and mitochondrial RNA content, following established guidelines for scRNA-seq data processing.

The average expression of the top 25 cell-type-specific genes from the scRNA-seq reference was calculated for each locus, constructing a signature score matrix. This matrix was analyzed using the get_enrichment_matrix and enrichment_analysis functions from the ‘Cottrazm**’** package to generate an enrichment scoring matrix, supporting subsequent analysis of cellular composition and providing insights into the relative abundance of each cell type within tissue spots.

Cellular enrichment across different tissue spots was visualized using the SpatialFeaturePlot function from the ‘Seurat**’** package, where higher enrichment scores were represented by darker colors, indicating increased abundance of a given cell type. Tissue regions were categorized into malignant, normal, and mixed groups based on the relative abundance of malignant cells, with thresholds defined by their enrichment scores. Differences in gene expression among these groups were assessed using the Wilcoxon rank-sum test.

Based on the deconvolution results, the cell type with the highest abundance in each microregion was identified and visualized using the SpatialDimPlot function. Additionally, the SpatialFeaturePlot was used to depict the expression landscape of the key gene across the tissue microregions. Correlations between cell abundance, other cell types, and the key gene expression were computed using Spearman correlation analysis and visualized with the ‘linkET**’** package, revealing interactions and dependencies between key gene and specific cellular populations.

Similarly, in the pan-cancer analysis, each microregion in the spatial transcriptomic slices was labeled by its predominant cell type. For instance, regions dominated by malignant cells were labeled as “malignant,” while those with endothelial cells as the majority were labeled as “endothelial.” The average expression of the key gene across all cell types in each slice was calculated and standardized using the scale function for z-score normalization. Heatmaps generated with the ‘pheatmap’ package visualized the normalized expression levels, highlighting the spatial distribution of the key gene across cell types.

### Statistical analysis

Statistical analyses were conducted using R-4.1.2 software and its associated packages. Wilcoxon rank sum tests were applied to evaluate differences in gene expression between tumor, normal, and mixed regions (spatial spots with ≥ 20% malignant + ≥ 20% non-malignant signals) identified in spatial transcriptomic analyses. For single-cell transcriptomic data, the Kruskal-Wallis H-test was used to compare the expression of key gene across different cell types, and Spearman correlation coefficients were calculated to assess associations with immune cell proportions and gene expression levels. Kaplan-Meier survival curves and Log-Rank tests were performed to evaluate survival differences between high-risk and low-risk patient groups. Additionally, univariate and multivariate Cox regression analyses were conducted to identify independent predictors of overall survival. Hazard ratios and 95% confidence intervals were calculated to quantify the prognostic impact of key variables. A P-value < 0.05 was considered statistically significant.

**Fig. 1:**
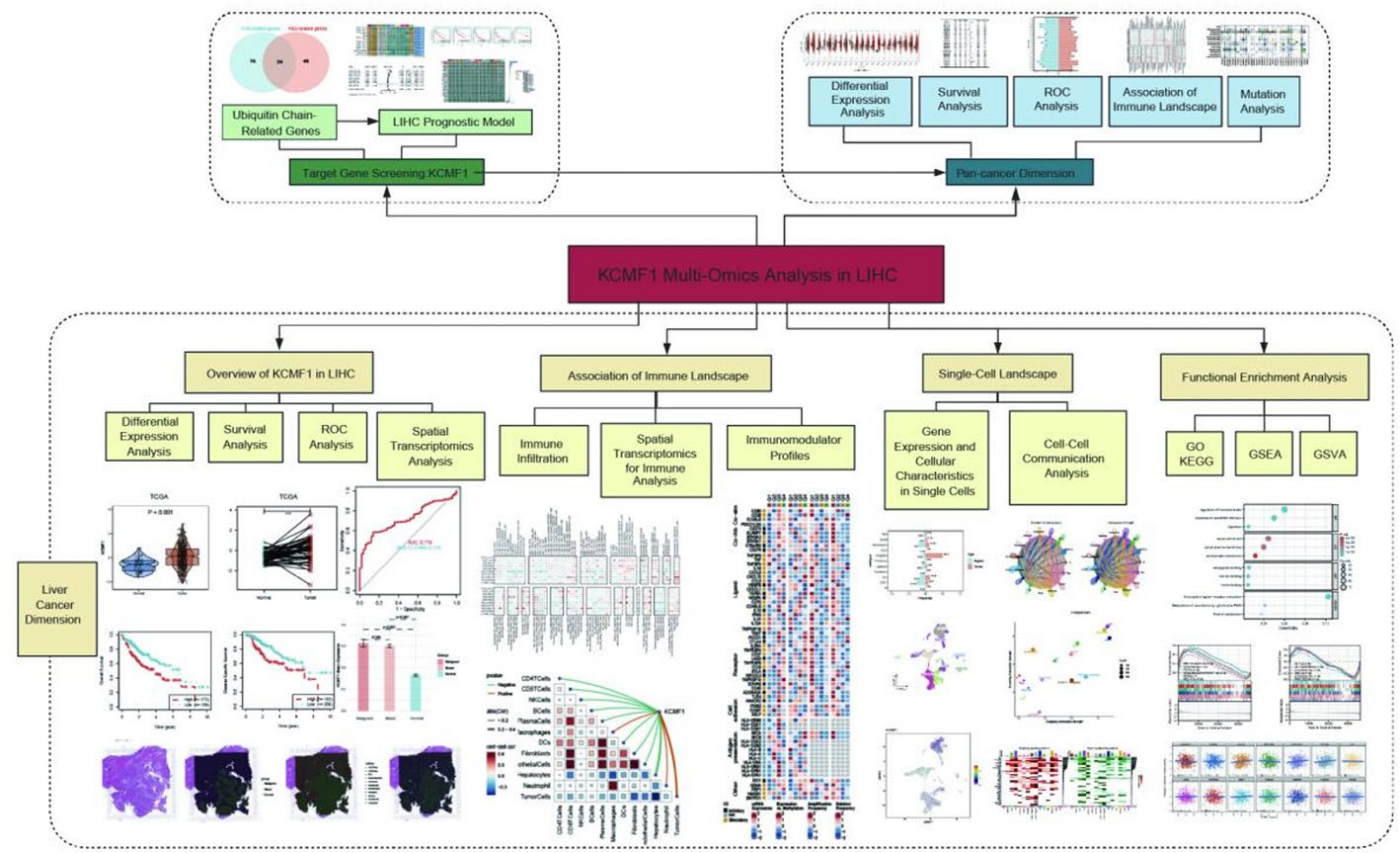
Flowchart of the study design

## Result

### Prognostic model validation and key gene identification

**Fig. 2:**
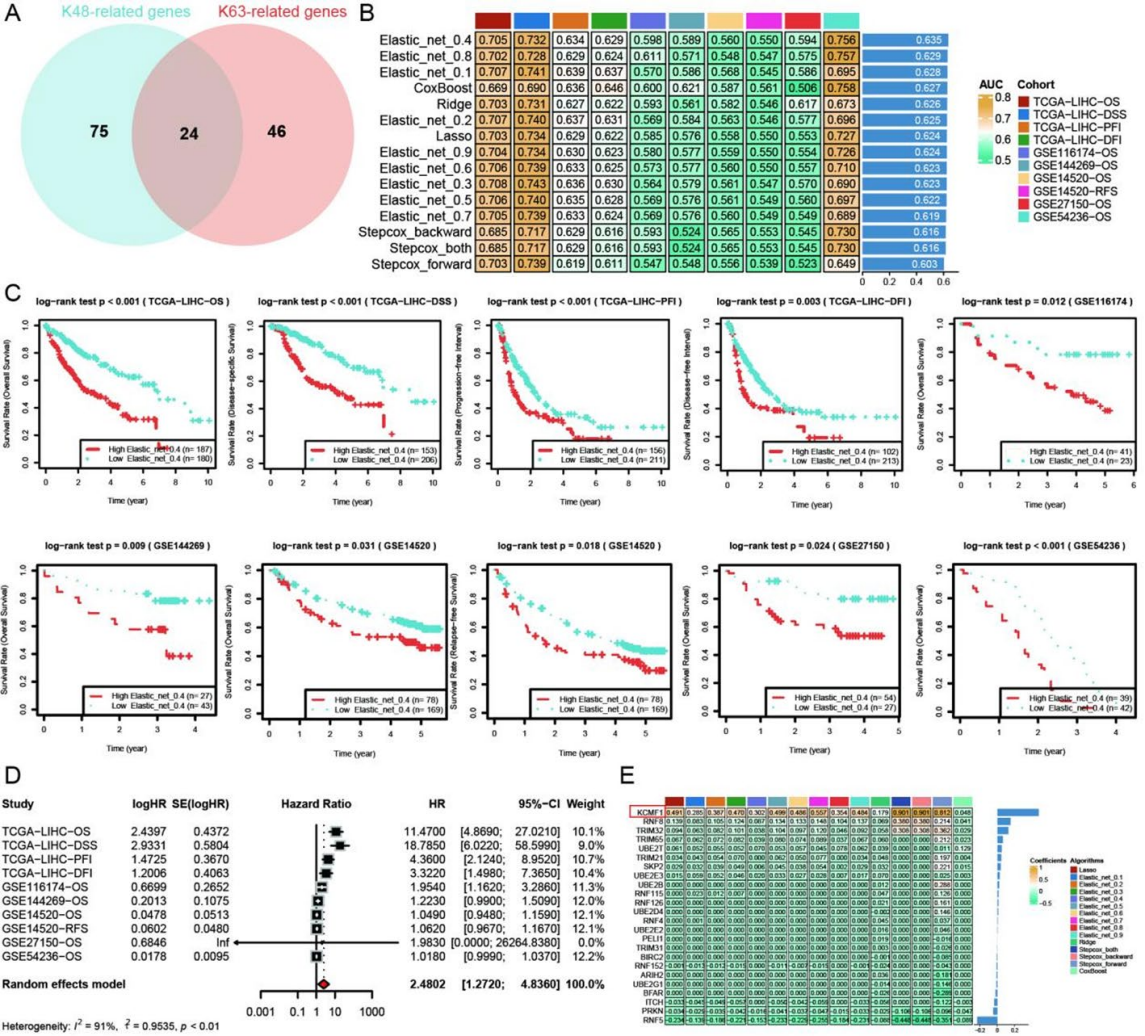
Prognostic evaluation of intersecting genes from K48- and K63-ubiquitin chains. **A** Venn diagram showing 24 IGS from K48- and K63-ubiquitin chain-associated gene sets. **B** Average AUC values at 1, 3, and 5 years for model evaluation using multiple algorithms. **C** Kaplan-Meier survival analysis indicating significantly worse outcomes in high-risk groups across 10 cohorts. **D** Univariate Cox regression and meta-analysis based on the Elastic_net_0.4 model. **E** Heatmap of regression coefficients illustrating the contributions of 24 genes, with KCMF1 identified as a key contributor

The comprehensive study design is depicted in Fig. 1. As shown in Fig. 2A, we identified 24 intersecting genes from the K48- and K63-ubiquitin chain-associated gene sets, collectively referred to as the IGS. Detailed information on K48- and K63-related genes is provided in Supplementary Table 1. To determine the best-performing model, we calculated the C-index (or AUC) for each algorithm across all validation cohorts. The Elastic Net model with α = 0.4 was selected as the final prognostic model because it exhibited the highest average C-index and consistent stability across the majority of datasets. To develop a robust prognostic model, we evaluated multiple algorithms using average AUC values at 1, 3, and 5 years. Among these, the Elastic_net_0.4 model was identified as the best-performing algorithm due to its consistently superior AUC values across all time points (Fig. 2B).

Kaplan-Meier survival analysis further demonstrated the model’s clinical relevance, with high-risk groups exhibiting significantly worse outcomes than low-risk groups in 10 survival cohorts across six datasets (Fig. 2C). Additionally, univariate Cox regression and meta-analysis of risk scores derived from the Elastic_net_0.4 model confirmed its robustness as a prognostic factor (Fig. 2D). These results highlight the model’s high predictive accuracy and generalizability, establishing it as a valuable tool for clinical prognostic evaluation.

A heatmap of regression coefficients revealed the relative contributions of the 24 genes across different models, with potassium channel modulatory factor 1 (KCMF1) consistently ranked as the top contributor (Fig. 2E). KCMF1 was subsequently identified as the key gene for further analysis.

### LIMA1 highlights KCMF1’s clinical relevance in liver cancer

**Fig. 3:**
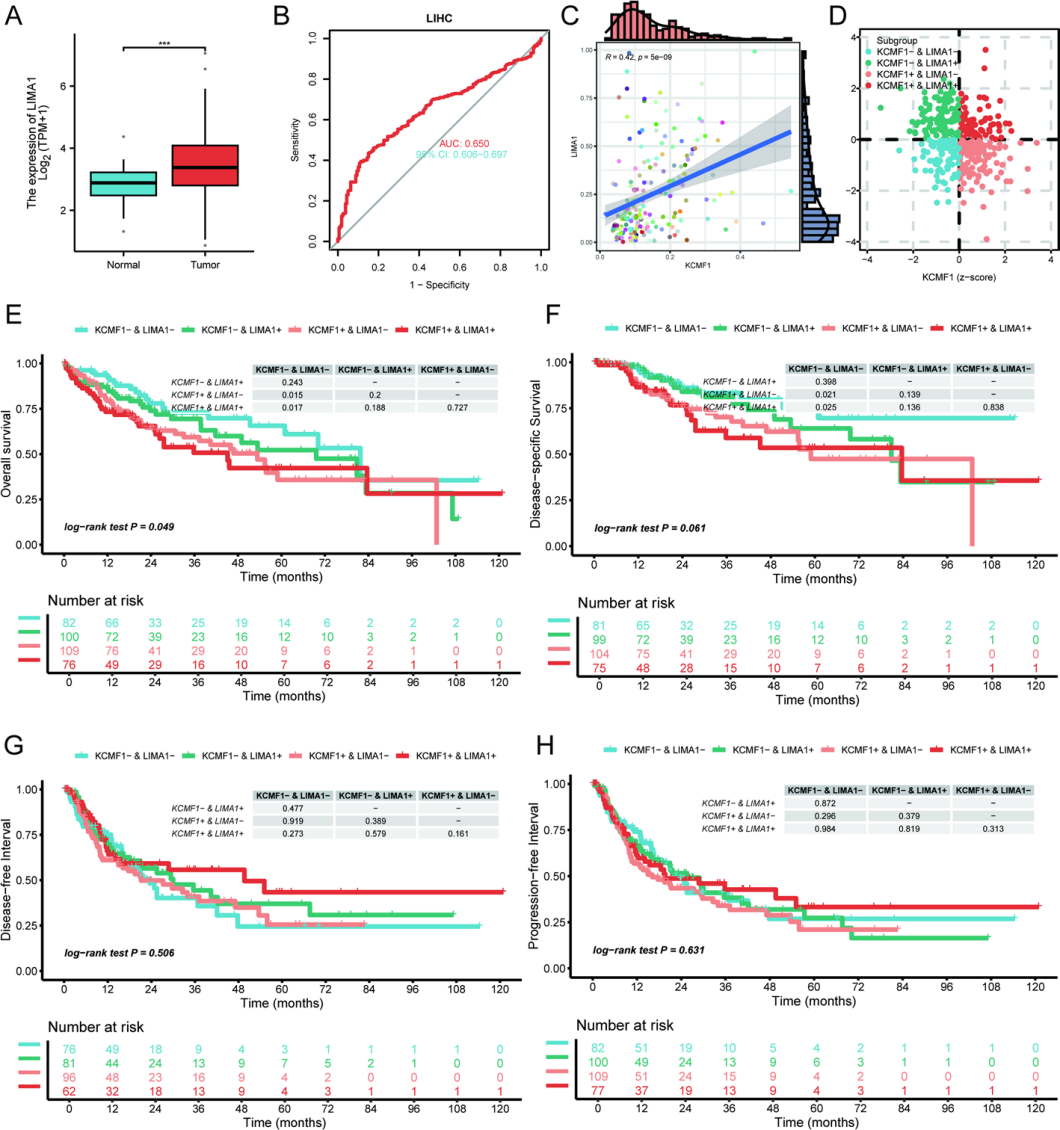
Relationship between LIMA1 and KCMF1 in HCC. **A** LIMA1 mRNA expression in tumor and normal tissues. **B** ROC curve analysis evaluating the diagnostic accuracy of LIMA1 expression. **C** Correlation analysis between LIMA1 and KCMF1 expression levels. **D** Subgroup classification based on z-score normalized expression levels of LIMA1 and KCMF1. **E**–**H** Kaplan-Meier survival analysis comparing OS, DSS, PFI, and DFI among the four subgroups defined by LIMA1 and KCMF1 expression levels. ***p < 0.001

Building on our previous findings that LIMA1 plays a critical role in liver cancer through ubiquitination modification, we explored its relationship with KCMF1 to better understand their combined clinical impact. LIMA1 mRNA expression analysis revealed significantly higher levels in tumor tissues compared to normal tissues (*P* < 0.001; Fig. 3A), with moderate diagnostic accuracy (AUC = 0.650; Fig. 3B). Correlation analysis showed a significant positive association between LIMA1 and KCMF1 expression (*R* = 0.42, *P* = 5e-09; Fig. 3C), suggesting potential biological interplay. Although relevance does not directly equate to functional synergy, combined with the subsequent immunomodulatory role of KCMF1, these findings suggest that the two may jointly participate in key pathways in hepatocellular carcinoma.

To further investigate their combined impact, we categorized samples into four subgroups based on the z-score normalization of LIMA1 and KCMF1 expression levels: LIMA1+&KCMF1+, LIMA1-&KCMF1+, LIMA1-&KCMF1-, and LIMA1+&KCMF1- (Fig. 3D). Kaplan-Meier survival analysis demonstrated that patients in the LIMA1+&KCMF1 + group had significantly worse OS (*P* = 0.017) and DSS (*P* = 0.025) compared to the LIMA1-&KCMF1- group, while no significant associations were observed for PFI or DFI (Fig. 3E-H). With their combined high expression strongly associated with poorer survival outcomes, we hypothesized that LIMA1 and KCMF1 synergistically promote liver cancer progression.

Additionally, pan-cancer TCGA data show KCMF1 is markedly up-regulated in tumors versus normal tissues (*P* < 0.05, Fig. S1A), especially in CESC, CHOL, KIRC, LIHC, LUAD and LUSC. High expression predicts poorer OS, DFI, DSS and PFI (HR > 1, Fig. S1B–E) and is validated in external cohorts (Fig. S1F), with ROC confirming diagnostic accuracy (Fig. S1G). Immune analyses reveal negative correlations with infiltrating cells, immune genes and immunogenicity/DNA-damage scores (Fig. S1H–J), indicating immune-evasive function. TP53 nonsense mutations may fuel its dysregulation (Fig. S1K, L), while single-cell and spatial transcriptomics place KCMF1 within malignant microregions (Fig. S2A, B), highlighting its microenvironment-shaping role.

### KCMF1 overexpression in HCC Based on transcriptomic and spatial analyses

**Fig. 4:**
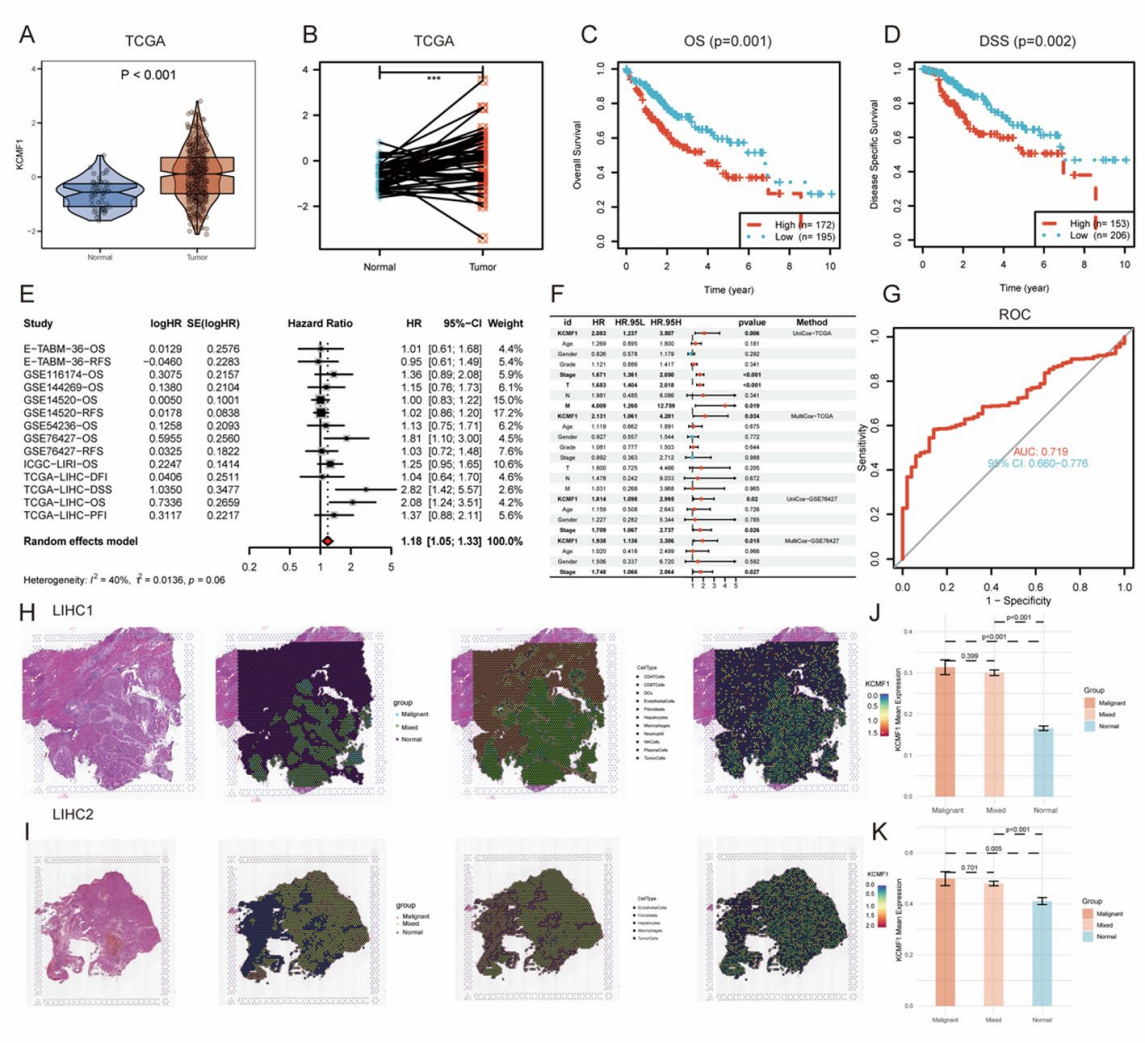
Expression profile and clinical relevance of KCMF1 in HCC. **A** mRNA expression of KCMF1 in HCC tissues compared to normal tissues in the TCGA dataset. **B** Pairwise comparison of KCMF1 expression between paired tumor and adjacent normal samples in TCGA. **C**, **D** Kaplan-Meier survival analysis showing poorer OS and DSS in patients with high KCMF1 expression in the TCGA cohort. **E** Meta-analysis of univariate Cox regression across multiple HCC datasets confirming KCMF1 as a risk factor for poor prognosis. **F** Multivariate Cox regression analysis demonstrating KCMF1 as an independent prognostic factor in HCC. **H**, **I** The spatial distribution of tumor microenvironment and KCMF1 expression in two HCC samples. **J**, **K** Differences in KCMF1 expression between malignant and normal regions in spatial transcriptomic analysis. *P < 0.05; **P < 0.01; ***P < 0.001

To independently evaluate KCMF1, we next explored its expression profile in HCC from two perspectives: transcriptomic and spatial transcriptomic analyses. mRNA analysis revealed significantly higher KCMF1 expression in HCC tissues compared to normal tissues in the TCGA dataset (*P* < 0.001; Fig. 4A). Pairwise comparisons further confirmed this trend in paired tumor and adjacent normal samples (*P* < 0.001; Fig. 4B). Analysis of six independent GEO datasets supported these findings, showing consistently elevated KCMF1 expression in HCC tissues (E_TABM_36: *P* = 0.01; GSE14520: *P* < 0.001; GSE39791: *P* < 0.001; GSE54236: *P* = 0.001; GSE112790: *P* < 0.001; GSE144269: *P* < 0.001) (Fig. S3A). Kaplan-Meier survival analysis in the TCGA cohort revealed that high KCMF1 expression was associated with significantly poorer OS (*P* = 0.001) and DSS (*P* = 0.002) (Fig. 4C, D). No significant associations were observed for PFI or DFI (Fig. S3B). Meta-analysis of univariate Cox regression across multiple HCC datasets confirmed KCMF1 as a risk factor for poor prognosis, with a pooled HR of 1.18 (95% CI 1.05–1.33) and moderate heterogeneity (I² = 40%, *P* = 0.06) (Fig. 4E). Multivariate Cox regression further demonstrated that KCMF1 is an independent prognostic factor, irrespective of clinical stage and other variables (Fig. 4F). ROC curve analysis highlighted the diagnostic potential of KCMF1, showing an AUC of 0.719 (95% CI 0.660–0.776) in distinguishing HCC patients from normal controls (Fig. 4G).

These findings establish KCMF1 as a clinically significant biomarker in HCC. Its association with poor prognosis and strong diagnostic accuracy highlights its potential for risk stratification and clinical decision-making. Building on these transcriptomic findings, we further examined the spatial distribution of KCMF1 in the tumor microenvironment using spatial transcriptomic analysis. In two HCC samples (HCC1 and HCC2), KCMF1 expression showed a strong spatial overlap with mixed regions, which represent areas containing signals from multiple cell types (Fig. 4H, I). This overlap suggests that KCMF1 may play a role in regions where tumor cells interact with surrounding non-tumor cells, potentially reflecting dynamic crosstalk between malignant and stromal or immune cells. Furthermore, after classifying malignant and normal regions, we observed that KCMF1 expression was significantly higher in malignant regions compared to normal regions, consistent with transcriptomic data from TCGA and GEO (HCC1: *P* < 0.001; HCC2: *P* = 0.005) (Fig. 4J, K).

Together, these results from transcriptomic and spatial analyses confirm that KCMF1 is overexpressed in HCC, particularly in tumor-associated regions, highlighting its potential role in tumor progression and microenvironmental dynamics.

### KCMF1 modulates the immune landscape in HCC

To investigate the role of KCMF1 in the immune microenvironment of HCC, we analyzed its association with immune infiltration using both transcriptomic and spatial transcriptomic data.

**Fig. 5:**
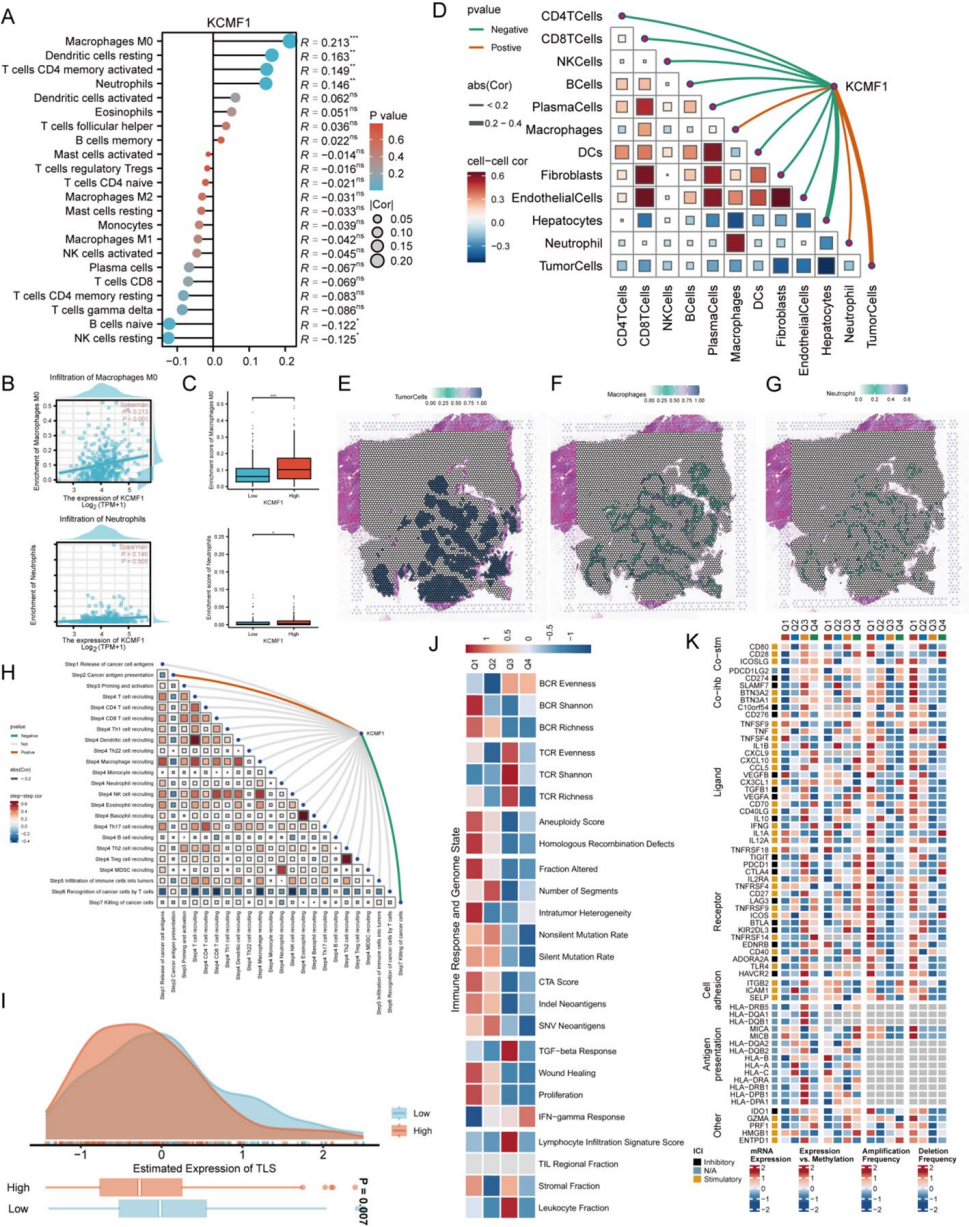
Immune microenvironment analysis of KCMF1 in HCC. **A** Correlation between KCMF1 expression and immune cell infiltration, assessed using the CIBERSORT algorithm. **B** Scatterplots showing significant positive correlations between KCMF1 expression and immune scores for macrophages M0 and neutrophils. **C** Comparison of immune scores for macrophages M0 and neutrophils between high- and low-KCMF1 expression groups. **D** Spearman correlations between KCMF1 expression and microenvironmental components at spatial resolution in HCC1. **E**–**G** Correlations between KCMF1 expression and malignant macrophages M0, and neutrophils cells in the HCC1 sample, based on spatial transcriptomics. **H** Correlation of KCMF1 expression with steps in the cancer immunity cycle, analyzed using the TIP algorithm. **I** Comparison of TLS scores between high- and low-KCMF1 expression groups, evaluated using the EASIER tool. **J** Associations between KCMF1 expression and immunogenicity as well as DNA damage scores in HCC. **K** Correlations between KCMF1 expression and immunomodulatory molecules in HCC. *P < 0.05; **P < 0.01; ***P < 0.001

Using the CIBERSORT algorithm, we found that KCMF1 expression was positively correlated with macrophages M0, dendritic cells resting, T cells CD4 memory activated, and neutrophils, while negatively correlated with B cells naive and NK cells resting (Fig. 5A). These results were further confirmed by Spearman correlation analysis using multiple algorithms, yielding consistent findings (Fig. S3C).

To further explore KCMF1’s association with specific immune cells, we focused on macrophages M0 and neutrophils. Scatterplot analyses revealed a significant positive correlation between KCMF1 expression and immune scores for both macrophages M0 (*R* = 0.213, *P* < 0.001) and neutrophils (*R* = 0.146, *P* = 0.005) (Fig. 5B). Patients in the high KCMF1 expression group exhibited significantly higher immune scores for these cells compared to the low-expression group (Macrophages M0: *P* < 0.001; Neutrophils: *P* < 0.05) (Fig. 5C). These findings suggest that KCMF1 is associated with an enrichment of immune cell populations favoring tumor progression, particularly M0 macrophages that represent the precursor pool for subsequent polarization into pro-tumor M2-like TAMs, in the HCC microenvironment.

In the spatial transcriptomic analysis, the Spearman correlation between KCMF1 expression and microenvironment components was visualized at spatial resolution (Fig. 5D, Fig. S3D). KCMF1 expression was strongly positively correlated with malignant tumor cell abundance, consistent with previous gene localization results (Fig. 5E, Fig. S3E). Note: HCC2 exhibited an inverse correlation between KCMF1 and macrophage abundance, likely reflecting intra-tumoral spatial heterogeneity. This discrepancy may reflect sample-specific heterogeneity, regional expression variation, or technical noise in spatial deconvolution. In the HCC1 sample, KCMF1 expression was positively correlated with both macrophages and neutrophils, aligning with transcriptomic findings (Fig. 5F, G). However, in the HCC2 sample, KCMF1 expression showed a significant negative correlation with macrophages. Larger cohorts are needed to determine whether KCMF1 exhibits spatially distinct immune-regulatory roles across tumor subregions or HCC subtypes. While the findings suggest that KCMF1 generally promotes the presence of macrophages in tumor regions, the variation observed in HCC2 highlights the complexity of these interactions and warrants further investigation. Additionally, KCMF1 expression was significantly negatively correlated with several anti-tumor cell types, including immune cells (CD4 + T cells, CD8 + T cells, NK cells, B cells, plasma cells, dendritic cells) as well as stromal cells (fibroblasts, endothelial cells) and hepatocytes in HCC1. Consistent negative correlations were observed in HCC2 across multiple cell types, including CD8 + T cells, fibroblasts, endothelial cells, and hepatocytes, reinforcing the potential role of KCMF1 in suppressing anti-tumor immune responses.

To further evaluate the role of KCMF1 in anti-tumor immunity, we employed the Tracking Tumor Immunophenotype (TIP) algorithm. TIP analysis showed that KCMF1 was positively correlated with step 2 (cancer antigen presentation) but negatively correlated with step 7 (cancer cell killing) of the cancer immunity cycle (Fig. 5H). Using the EASIER tool, which predicts immune therapy outcomes based on RNA-seq data, we found that the tertiary lymphoid structure (Tertiary lymphoid structure, TLS) score was significantly higher in the KCMF1 low-expression group (*P* = 0.007; Fig. 5I), indicating that lower KCMF1 expression may be associated with a more favorable immune microenvironment.

Finally, we examined the immune landscape comprehensively by analyzing the relationship between KCMF1 expression and immunogenicity and DNA damage scores (Fig. 5J). Furthermore, the association between KCMF1 expression and immunomodulatory molecules was investigated to map a detailed immunity landscape for KCMF1 in HCC (Fig. 5K, Fig. S3F).

### KCMF1 is highly expressed in malignant cells and monocyte/macrophage lineages

**Fig. 6:**
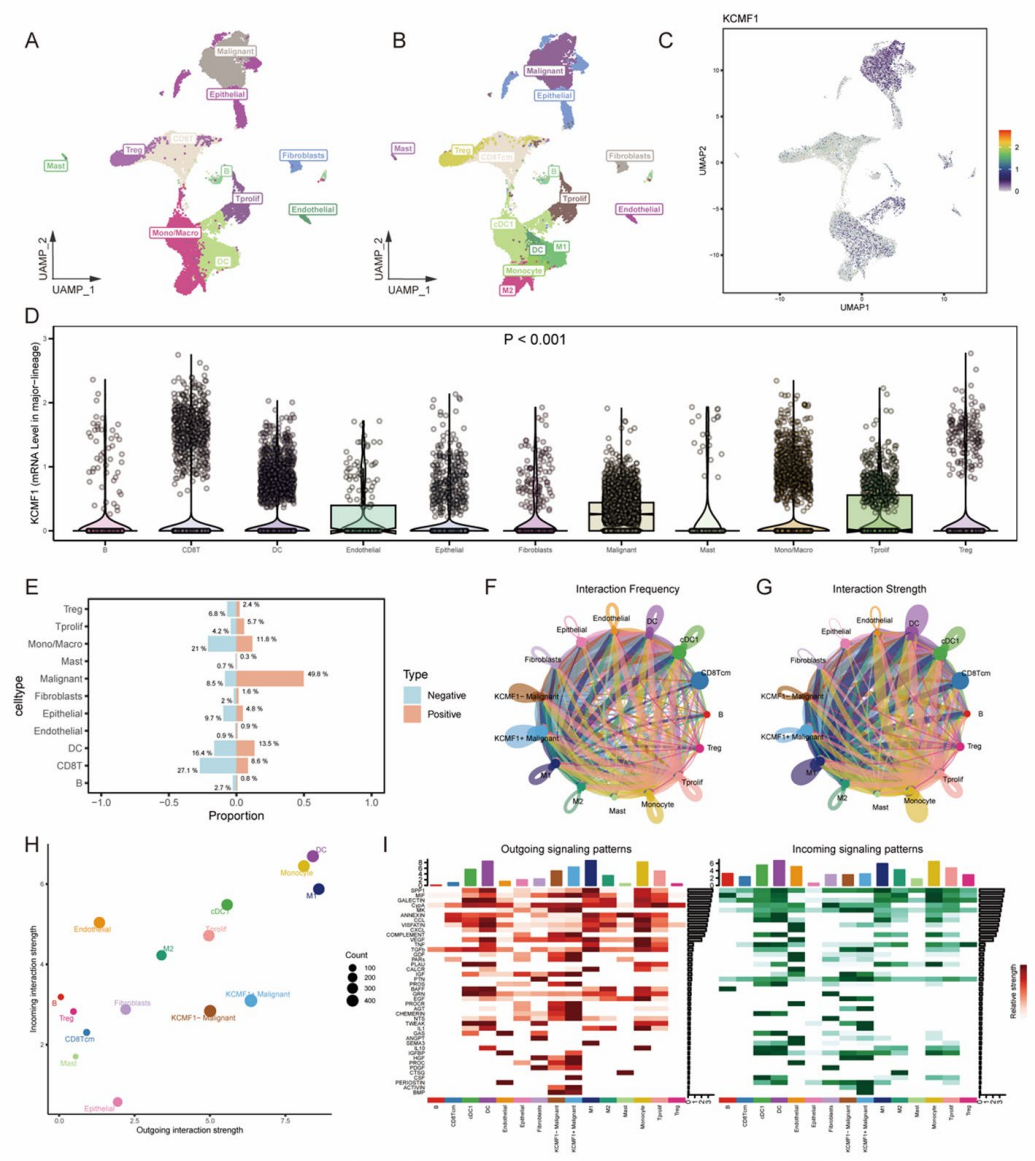
Cellular distribution and communication patterns of KCMF1 in the TME. **A**, **B** UMAP visualization showing major and fine cellular lineages in the GSE166635 dataset. **C** Visualization of KCMF1 expression across different cell populations. **D** Expression levels of KCMF1 across various cell lineages. **E** Comparison of cell type proportions between KCMF1-positive and KCMF1-negative groups. **F**, **G** Visualization of cell-cell interaction frequency and intensity in the tumor microenvironment. **H** Outgoing and incoming interaction strengths of different cell types in the tumor microenvironment. **I** Signaling patterns of key pathways in outgoing and incoming interactions across cell types

To investigate the cellular distribution of KCMF1, we conducted single-cell analysis using the GSE166635 dataset. Dimensionality reduction and visualization were performed using UMAP, which effectively distinguished different cell populations based on their gene expression profiles. This analysis identified both major and fine cellular lineages (Fig. 6A, B). Notably, KCMF1 expression was predominantly observed in malignant cells, monocytes/macrophages (mono/macro), and DCs, as illustrated in Fig. 6C. These findings were further validated using the GSE146115 and GSE146409 datasets, which showed consistent patterns of KCMF1 expression in the same cell types (Fig. S4A, B).

To further explore the cellular specificity of KCMF1, we assessed its expression across different cell lineages. While KCMF1 was detected in all cell types, its expression was particularly elevated in malignant cells, mono/macro, and DCs (Fig. 6D). We next compared the proportions of each cell type between the KCMF1-positive and KCMF1-negative groups. The results revealed that the KCMF1-positive group exhibited significantly higher proportions of malignant cells and mono/macro compared to the KCMF1-negative group (Fig. 6E). We conducted a comprehensive cell-cell communication analysis to investigate the role of KCMF1 in the TME, considering two dimensions: Count, representing interaction frequency, and Weight, reflecting interaction intensity (Fig. 6F, G). In the initial analysis, DCs, macrophages M1, and monocytes exhibited the highest interaction frequency and intensity among all cell types, underscoring their critical roles as communication hubs within the TME (Fig. S4C, D).

Further analysis revealed that DCs, macrophages M1, and monocytes showed prominent strengths for both outgoing and incoming interactions (Fig. 6H). In outgoing signaling patterns, these cells demonstrated significant activity in the VEGF and TGFβ pathways, highlighting their involvement in angiogenesis and immune regulation (Fig. 6I). Concurrently, these cells exhibited strong incoming signaling activity in the TNF pathway, emphasizing their roles in responding to inflammatory signals.

Additionally, KCMF1-positive malignant cells exhibited significant enrichment across multiple signaling pathways, both in outgoing and incoming signaling patterns. Signal intensity was quantified by the CellChat ‘weight’ parameter, which integrates ligand-receptor pair expression and downstream pathway activity. Their signal intensity was markedly higher than that of KCMF1-negative malignant cells, indicating a more active role in intercellular communication. This contrast highlights the potential of KCMF1-positive cells as key contributors to tumor progression and as actionable targets for therapy. Lastly, ligand-receptor analysis revealed intricate signaling networks between different cell types in the tumor microenvironment, highlighting interactions that could serve as therapeutic or regulatory targets (Fig. S4E, F).

### KCMF1-driven pathways and tumor phenotypes

**Fig. 7:**
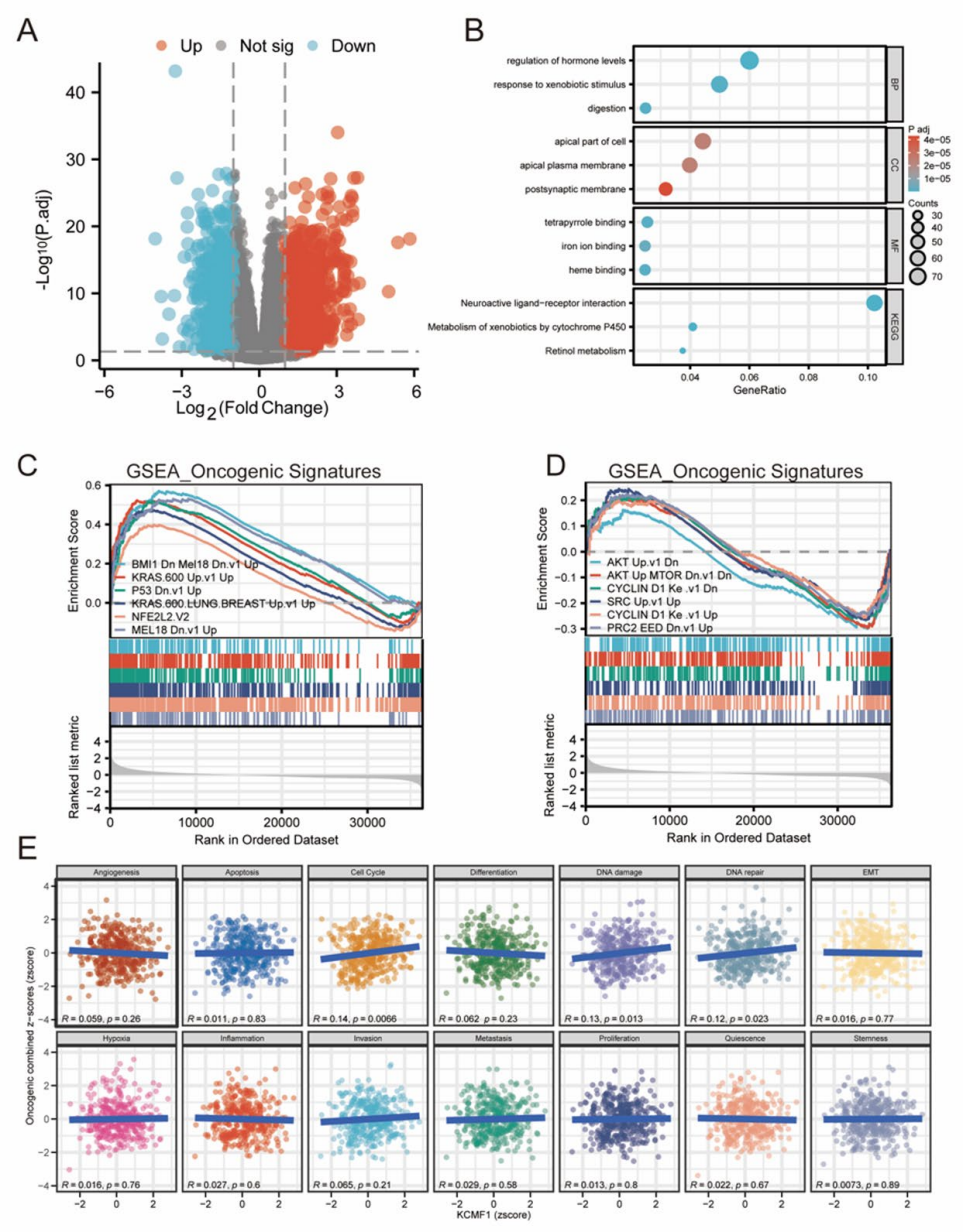
Functional analysis of KCMF1 in HCC. **A** Differential expression analysis of HCC samples grouped by KCMF1 expression, showing the identification and visualization of DEGs. **B** GO and KEGG enrichment analyses of DEGs. **C**, **D** GSEA analysis of oncogenic signatures using unfiltered genes, presenting upregulated and downregulated pathways. **E** GSVA analysis of the association between KCMF1 expression and tumor phenotypes

To investigate the biological function of KCMF1 in HCC, we first divided the HCC samples into two groups based on the median expression of KCMF1 and performed differential expression analysis. This analysis identified both upregulated and downregulated DEGs. Using the thresholds of |log2 Fold Change| > 1 and *P* < 0.05, we screened the DEGs and visualized the results (Fig. 7A).

Subsequently, GO and KEGG enrichment analyses of the DEGs were performed (Fig. 7B). The GO analysis revealed enrichment in biological processes (BP) such as regulation of hormone levels, response to xenobiotic stimulus, and digestion; cellular components (CC) such as the apical part of the cell, apical plasma membrane, and postsynaptic membrane; and molecular functions (MF) such as tetrapyrrole binding, iron ion binding, and heme binding. The KEGG analysis highlighted pathways including neuroactive ligand-receptor interaction, metabolism of xenobiotics by cytochrome P450, and retinol metabolism, suggesting that KCMF1 may influence metabolic and signaling pathways relevant to tumor progression. Although causal links await functional validation, bioinformatic prediction suggests that dysregulated bile-acid metabolism may activate the FXR–S1P axis, thereby enhancing tumor-cell proliferation and immune evasion.

Further, GSEA analysis of the Oncogenic Signatures gene set using unfiltered genes revealed significant upregulation of pathways such as BMI1 Dn Mel18 Dn.v1 Up, KRAS.600 Up.v1 Up, and SRC Up.v1 Up, which are closely associated with tumorigenesis and cancer progression. In contrast, pathways such as AKT Up MTOR Dn.v1 Dn and CYCLIN D1 Ke.v1 Dn were significantly downregulated, potentially indicating mechanisms that counteract tumor progression (Fig. 7C, D).

Finally, gene set variation analysis (GSVA) analysis revealed that KCMF1 expression was significantly correlated with tumor phenotypes such as cell cycle (*R* = 0.14, *P* = 0.0066) and DNA damage (*R* = 0.13, *P* = 0.013) (Fig. 7E). These results underscore the central role of KCMF1 in modulating tumor biology, potentially through its impact on key pathways and phenotypes related to cancer progression.

## Discussion

Hepatocellular carcinoma is the most common form of primary liver cancer, characterized by high mortality and recurrence rates [34]. Despite advances in early detection and treatment, the prognosis for HCC remains poor, with a five-year survival rate below 20% [35]. Understanding the molecular mechanisms underlying HCC is critical for developing more effective therapeutic targets and identifying promising prognostic biomarkers. Ubiquitination has recently attracted significant attention in cancer biology [7–9]. K48 and K63 ubiquitin chains, the most common types of ubiquitination modifications, are involved in protein degradation and signal transduction, respectively [15]. Genes associated with these ubiquitin chains may play essential roles in tumor development and progression. Based on these, we identified 24 intersecting genes from K48- and K63-related gene sets as candidate prognostic markers.

Among the candidate genes, KCMF1 emerged as a key gene. Previous studies have confirmed that KCMF1 may be associated with the development of renal clear cell carcinoma, colon cancer, and pancreatic cancer [36–38]. However, its expression patterns, clinical significance, and molecular mechanisms in HCC have not been systematically investigated. Through constructing the Elastic_net_0.4 prognostic model, we validated the robust predictive performance of KCMF1 over multiple time points (1 year, 3 years, and 5 years in terms of AUC), confirming its clinical predictive value.

Transcriptomic analysis revealed that KCMF1 expression was significantly elevated in HCC tumor tissues compared to normal tissues and was associated with worse prognosis, including overall survival and disease-specific survival. Multivariate Cox regression analysis confirmed that KCMF1 is an independent prognostic factor, while ROC curve analysis demonstrated its strong diagnostic performance in distinguishing tumor tissues from normal tissues. Additionally, immune infiltration analysis showed that KCMF1 expression was positively correlated with M0 macrophage enrichment (Spearman *R* = 0.213, *P* < 0.001) and neutrophil infiltration (Spearman *R* = 0.146, *P* = 0.005), and negatively correlated with anti-tumor immune cells, including CD8 + T cells and NK cells (Fig. 5B). We further calculated M1 and M2 macrophage scores and analyzed their correlation with KCMF1 expression. These results are presented in Supplementary Fig. 5. Notably, KCMF1 showed no significant direct correlation with polarized M1 (Spearman *R* = -0.042, *P* = 0.415) or M2 (Spearman *R* = -0.031, *P* = 0.553) phenotypes. This discrepancy—significant association with M0 but not with M1/M2—suggests that KCMF1 may indirectly regulate macrophage polarization by modulating monocyte recruitment and precursor availability, rather than directly controlling polarized states. Our observation that KCMF1-associated M0 enrichment indicates precursor availability for M2 polarization aligns with and extends the established TAM paradigm. Mantovani and colleagues demonstrated that tumor-associated macrophages originate from circulating Ly6C+ monocytes recruited to the tumor site, where local cytokines drive their differentiation into pro-tumor M2-like cells [39]. Our data suggest that KCMF1-mediated ubiquitination networks may function as a “molecular rheostat” modulating the kinetics of M2 polarization, integrating post-translational control into the classical TAM differentiation model. When combined with single-cell and spatial transcriptomic analyses, these findings further validated the potential roles of KCMF1 in shaping the TME.

KCMF1 likely promotes HCC progression through multiple mechanisms, including immune regulation, metabolic reprogramming, signaling pathway synergy, and dynamic intercellular interactions. Three mechanistic axes of KCMF1-driven HCC progression are proposed: [1] Immune-microenvironment remodelling. Bulk, single-cell and spatial data consistently show that high KCMF1 expression enriches M0 macrophages and neutrophils while reducing CD8 + T and NK cells (Fig. 5B). CellChat analysis further indicates that KCMF1-positive malignant cells intensify outgoing VEGF and TGFβ signals, converting these myeloid populations into an immunosuppressive hub. Consequently, the cancer-immunity cycle is blocked at the cancer-cell killing step (Fig. 6). The VEGF/TGFβ axes identified in our analysis represent established HCC therapeutic targets. The SHARP trial established sorafenib as first-line therapy targeting VEGF signaling [40], yet resistance mechanisms involving TGFβ-mediated immune exclusion remain incompletely understood. Our data position KCMF1 at the nexus of these pathways, suggesting that ubiquitination modulation may coordinate stromal activation and immune evasion, with implications for combined VEGF/TGFβ blockade strategies [2]. Metabolic reprogramming. GSEA and GSVA link KCMF1 to bile-acid metabolism, iron-ion binding and xenobiotic pathways. Dysregulated bile acids can modulate tumor progression and immune evasion by engaging host receptors such as FXR and S1PR2, which influence the tumor immune microenvironment and CD8⁺ T cell function [41, 42]. Meanwhile, iron overload exacerbates hepatic oxidative stress and injury, a process mitigated by FXR activation, suggesting a role for iron-induced reactive oxygen species (ROS) in promoting cellular damage [43]. KCMF1 may therefore couple metabolic rewiring to the above immune escape programme [3]. Oncogenic-signal synergy and KCMF1 mechanistic context. KCMF1-high tumors display activated KRAS, SRC and BMI1 signatures together with repressed AKT-mTOR feedback (Fig. 7C, D). This imbalance enhances proliferation, angiogenesis and therapy resistance, while the concurrent VEGF/TGFβ output amplifies stromal activation in the same lesion. Together, KCMF1 acts as a multi-functional orchestrator that synchronises immune suppression, metabolic adaptation and oncogenic signalling in HCC, rather than affecting a single pathway. Notably, KCMF1 lacks canonical HECT, RING, or UBL domains found in well-characterized HCC-associated E3 ligases such as MDM2, which promotes HCC through p53 degradation [44, 45], and FBXW7, which functions as a general tumor suppressor whose loss stabilizes oncogenic proteins including MYC and NOTCH1 [46]. This structural distinction suggests that KCMF1 likely operates as a scaffold or substrate adaptor rather than a catalytic enzyme, positioning it as a novel therapeutic node resistant to traditional E3-targeting strategies but amenable to disruption of protein-protein interactions.

Incomplete neutrophil polarization characterization. We acknowledge that N2 neutrophil scoring was not included in the current analysis. This omission stems from the limited availability of validated N2-specific gene signatures for hepatocellular carcinoma. Unlike the well-established M1/M2 paradigm for macrophages [39], neutrophil polarization nomenclature remains heterogeneous, with N1/N2 classifications primarily derived from preclinical models rather than human transcriptomic data [47]. Recent single-cell RNA sequencing studies have identified diverse neutrophil states in cancer, including pro-tumor subsets marked by VEGFA, CXCR4, and MMP9 expression [48, 49]; however, consensus signatures for N2 neutrophils comparable to the CIBERSORT LM22 gene set for macrophages are lacking [50]. Furthermore, the plasticity of neutrophil states and their rapid turnover in the tumor microenvironment complicate deconvolution-based scoring from bulk RNA-seq data [51]. Future studies incorporating single-cell transcriptomics or spatial transcriptomic profiling will be necessary to definitively establish KCMF1’s association with pro-tumor neutrophil subsets in HCC.

## Limitations

Despite these findings, this study has several limitations. First, our conclusions are primarily derived from integrative bioinformatic analyses of publicly available datasets; consequently, the lack of direct experimental validation precludes definitive establishment of causality between KCMF1 and ubiquitination chain dynamics in HCC progression. Second, the specific molecular mechanism by which KCMF1 regulates ubiquitination chain dynamics requires experimental validation to distinguish between direct enzymatic activity and scaffolding functions. Third, the spatial transcriptomic findings, though derived from clinically annotated samples, require validation in larger, multi-center cohorts to confirm the generalizability of KCMF1 as a diagnostic and prognostic biomarker across diverse etiological subtypes of HCC. Finally, the therapeutic implications of targeting KCMF1-mediated ubiquitination networks necessitate preclinical evaluation using both in vitro HCC models and in vivo patient-derived xenograft systems. Experimental validation of these hypotheses is currently underway. Nevertheless, by systematically integrating transcriptomic, spatial, and clinical data, this work establishes a framework for understanding ubiquitination dynamics in HCC and generates testable hypotheses that guide future experimental and translational investigation.

## Conclusion

In summary, our study provides the first comprehensive investigation of KCMF1 in HCC, revealing its potential roles in immune regulation, metabolic reprogramming, and tumor-stroma interactions. These findings highlight KCMF1 as a promising diagnostic and prognostic biomarker, and potential therapeutic target in HCC.

## Supplementary Material

Below is the link to the electronic supplementary material.

Supplementary Material 1.

## Data Availability

All data analysed in this study are publicly available. The raw RNA-sequencing and clinical data were downloaded from the following repositories with the indicated accession identifiers.GEO datasets: GSE87211: https://www.ncbi.nlm.nih.gov/geo/query/acc.cgi? acc=GSE87211; GSE54460: https://www.ncbi.nlm.nih.gov/geo/query/acc.cgi? acc=GSE54460; GSE70769: https://www.ncbi.nlm.nih.gov/geo/query/acc.cgi? acc=GSE70769. TCGA-LIHC: https://portal.gdc.cancer.gov/projects/TCGA-LIHC.ICGC-LIRI: https://dcc.icgc.org/releases/release_25/Projects/LIRI-JP.Additional HCC cohorts analysed: GSE116174:https://www.ncbi.nlm.nih.gov/geo/query/acc.cgi? acc=GSE116174; GSE144269: https://www.ncbi.nlm.nih.gov/geo/query/acc.cgi? acc=GSE14426; GSE14520: https://www.ncbi.nlm.nih.gov/geo/query/acc.cgi? acc=GSE14520; GSE54236: https://www.ncbi.nlm.nih.gov/geo/query/acc.cgi? acc=GSE54236. Single-cell RNA-seq datasets: GSE166635:https://www.ncbi.nlm.nih.gov/geo/query/acc.cgi? acc=GSE166635; GSE146115:https://www.ncbi.nlm.nih.gov/geo/query/acc.cgi? acc=GSE146115; GSE146409:https://www.ncbi.nlm.nih.gov/geo/query/acc.cgi? acc=GSE146409. Spatial transcriptomics datasets: The two 10× Visium HCC slides (HCC1 and HCC2) were obtained from the publicly available Mendeley Data repository (https://doi.org/10.17632/skrx2fz79n.1). All code used for analysis is available from the corresponding author upon reasonable request.

## Abbreviations

KCMF1: Potassium channel modulatory factor 1
HCC: Hepatocellular carcinoma
ROC: Receiver operating characteristic
AUC: Area under the curve
HR: Hazard ratios
CIs: Confidence intervals
SNV: Somatic single-nucleotide variant
TME: Tumor microenvironment
GO: Gene ontology
KEGG: Kyoto encyclopedia of genes and genomes
GSEA: Gene set enrichment analysis
DEG: Differently expressed genes
UMAP: Uniform manifold approximation and projection
CESC: Cervical squamous cell carcinoma and endocervical adenocarcinoma
CHOL: Cholangiocarcinoma
KIRC: Kidney renal clear cell carcinoma
KIRP: Kidney renal papillary cell carcinoma
LIHC: Liver hepatocellular carcinoma
LUAD: Lung adenocarcinoma
LUSC: Lung squamous cell carcinoma
UCEC: Uterine corpus endometrial carcinoma
BRCA: Breast invasive carcinoma
KICH: Kidney chromophobe
OS: Overall survival
PFI: Progression free interval
DSS: Disease-specific survival
DFI: Disease-free interval
DCs: Dendritic cells
TLS: Tertiary lymphoid structure
TIP: Tracking tumor immunophenotype
mono/macro: monocytes/macrophages
BP: Biological processes
CC: Cellular components
MF: Molecular functions
GSVA: Gene set variation analysis
TAMs: Tumor-associated macrophages
